# Identifying Soybean Germplasm with Tolerance to Dehydration and Salinity Stresses

**DOI:** 10.3390/plants15091355

**Published:** 2026-04-29

**Authors:** Yong-Bi Fu, Shanna M. Quilichini, Elroy R. Cober, Anfu Hou, Thomas D. Warkentin, Carolee Horbach, Thomas Judiesch

**Affiliations:** 1 Saskatoon Research and Development Centre, Agriculture and Agri-Food Canada, 107 Science Place, Saskatoon, SK S7N 0X2, Canada; carolee.horbach@agr.gc.ca; 2 Swift Current Research and Development Centre, Agriculture and Agri-Food Canada, Swift Current, SK S9H 3X2, Canada; shanna.quilichini@agr.gc.ca (S.M.Q.); thomas.judiesch@agr.gc.ca (T.J.); 3 Ottawa Research and Development Centre, Agriculture and Agri-Food Canada, Ottawa, ON K1A 0C6, Canada; elroy.cober@agr.gc.ca; 4 Morden Research and Development Centre, Agriculture and Agri-Food Canada, Morden, MB R6M 1Y5, Canada; anfu.hou@agr.gc.ca; 5 Department of Plant Sciences, University of Saskatchewan, 51 Campus Dr., Saskatoon, SK S7N 5A8, Canada; tom.warkentin@usask.ca

**Keywords:** germplasm characterization, soybean germplasm, PEG 6000, dehydration stress, salinity stress, abiotic stress tolerance, soybean breeding

## Abstract

Germplasm characterization can enhance the management and utilization of plant germplasm conserved in genebanks worldwide. This study was conducted to characterize 774 diverse soybean [*Glycine max* (L.) Merr.] accessions, mainly conserved at Plant Gene Resources of Canada (PGRC), through a laboratory seedling vigor test under polyethylene glycol (PEG)-induced dehydration stress and 72 selected accessions through a greenhouse salinity test. The PEG-based test identified 95 accessions that showed vigorous seedling growth in Petri dishes containing 20% (*w*/*v*) PEG 6000 solution. The salinity test revealed 58 accessions that produced total seed yields per plant ranging from 0.03 g to 1.47 g under severe salinity stress (EC_i_ 16.1 dS m^−1^). Six accessions originating from five countries displayed higher salt tolerance than the Canadian salt-tolerant cultivar OAC Ayton, but the latter still had the highest seed yield. One unique accession, CN29789, originating from China and named ‘Hei Nung No.18’, consistently showed high tolerance to both dehydration and salinity stresses and had vigorous root growth under severe salinity stress. These findings are significant, as they not only provide useful germplasm for soybean genetic improvement for abiotic stress tolerance but also demonstrate the value of characterizing plant germplasm conserved in a genebank for better utilization.

## 1. Introduction

Germplasm characterization can contribute to better management and utilization of germplasm collections conserved in genebanks worldwide [1,2]. However, many collections are not adequately characterized, as characterization efforts require substantial resources and funding support [1,3]. Many conserved germplasm accessions are documented with only basic germplasm descriptors such as passport data, and the accession-level information on conserved germplasm is largely lacking [4,5]. This problem is one of the major restraints of wider germplasm utilization [6,7], and consequently, the value of conserved germplasm is hardly realized [8]. Thus, more efforts are needed to characterize conserved germplasm for better utilization to fulfill the mandate of genebanks for sustainable agriculture [9,10,11].

Soybean [*Glycine max* (L.) Merr.] is one of the most important crops in the world [12,13], with 7.57 million tonnes produced in Canada in 2024 [14]. There is a recognized need to expand its production further north into the Canadian Prairies [15,16,17]. However, such expansion requires soybean germplasm that can be adapted to the heterogenous soil conditions of the Canadian Prairies, including the presence of salinity, for the development of productive cultivars with earlier maturity and increased protein concentration [18,19]. Soybeans grown in the Canadian Prairies can experience significant yield loss from soil salinity stress [20], as 30% of land across the Canadian Prairies, such as along the Red River Valley, displayed salinization before 2001 [21]. Salinity affects plant growth by imposing both osmotic and ionic stress [22]. Soil water potential is decreased with high salt concentrations, creating a physiological drought in plants. Research has revealed three major mechanisms for salt tolerance in plants, including chloride exclusion in roots, osmotic tolerance, and tissue tolerance [22,23]. Salt tolerance screening under field and greenhouse conditions is time-consuming, unpredictable, and unreliable [24]; yet, several salt-tolerant cultivars have been released in the USA [25].

Considerable efforts have been made to characterize soybean germplasm for drought tolerance in laboratories [26,27], greenhouses [28], and fields [29]. For example, Kakati et al. [26] simulated water stress using polyethylene glycol (PEG) 6000 in the laboratory to characterize 41 accessions and identified two accessions with better germination and root morphology. However, most characterizations screened less than 100 accessions due to the difficulty of environmental control and field water-control setup (e.g., [27,30]). Thus, much of the soybean germplasm conserved in genebanks remains poorly characterized for drought tolerance [31]. However, the early efforts to characterize USDA-ARS soybean germplasm under drought conditions (e.g., [32]) have greatly contributed to the advancement of soybean breeding programs for drought tolerance in the United States [33]. Several high-yielding cultivars with drought tolerance have been released in the USA [34].

Plant Gene Resources of Canada (PGRC; the Canadian national seed genebank at Saskatoon) maintains a soybean germplasm collection of 1031 accessions. These accessions were largely collected from Canadian soybean breeding programs over the past 50 years. Additionally, accessions with known early maturity were also acquired from the USDA-ARS soybean collection and the N.I. Vavilov All-Russian Institute of Plant Genetic Resources over the past 15 years. Since 2017, we have conducted several research activities to characterize the PGRC soybean collection. These research efforts have generated a large set of characterization data on the PGRC soybean germplasm in maturity, oil and protein concentration, and genetic distinctness [35,36]. However, no efforts have been made to characterize the PGRC soybean collection for tolerance to abiotic stresses. The objective of this study was to identify soybean germplasm with tolerance to dehydration and salinity stresses. Specifically, 774 accessions with sufficient seed quantities for research were screened in the laboratory for tolerance to polyethylene glycol (PEG)-induced dehydration. A set of soybean accessions were selected based on seedling growth under the PEG treatments and were subjected to a greenhouse salinity trial for an assessment of their tolerance to salinity stress. It was our hope that this study will generate a set of soybean germplasm with high tolerance to dehydration and salinity stresses for better utilization of the conserved germplasm to enhance soybean breeding.

## 2. Materials and Methods

### 2.1. Sample Selection and Acquisition

The study material included 763 accessions from the PGRC soybean collection with enough seed available for germplasm characterization, and 11 additional accessions of drought tolerance potential that were acquired by PGRC specifically for this study (Table S1). The 11 accessions were selected mainly through contributions from soybean breeders and information reported in published literature and, together with five accessions from the Canadian soybean breeding program in Ottawa, formed a control set of 16 accessions (Table S1). Soybean seed samples and their inventory data such as passport, country of origin, and accession description were acquired from the period of September 2022 to November 2024 from PGRC for public good research, following the Standard Material Transfer Agreement of the International Treaty on Plant Genetic Resources for Food and Agriculture (https://www.fao.org/plant-treaty/areas-of-work/the-multilateral-system/smta/en/; accessed 30 July 2025).

### 2.2. Laboratory Seedling Vigor Testing Under PEG-Induced Dehydration

For each accession, the testing procedure had three major steps: seed disinfection, germination testing under PEG-induced dehydration, and seedling vigor assessment. For seed disinfection, a PGRC protocol for soybeans was applied. Specifically, seeds were rinsed with tap water and then immersed in a mild soapy solution for 2 min, followed by a 1 min rinse with distilled water. Surface disinfection was performed using freshly prepared 10% (*v*/*v*) sodium hypochlorite solution for 1 min. Seeds were then rinsed thoroughly with distilled water and air dried on sterile filter paper.

For the seedling vigor test, water deficit was simulated using PEG 6000 (Thermo Fisher Scientific, Waltham, MA, USA) as an osmotic agent, and the general approach of Kakati et al. [26] was applied with modifications in PEG concentrations and seed number per replicate to accommodate large-scale germplasm screening and limited seed availability. Three dehydration treatments were applied: 0%, 10%, and 20% (*w*/*v*) PEG 6000 solutions prepared with deionized water (or PEG0, PEG10, and PEG20, respectively). The 0% PEG solution was deionized water only. For each accession and treatment, two replicate Petri dishes (9 cm diameter) were prepared, each containing 7 seeds evenly placed on two layers of Whatman No.1 filter paper. Each dish was filled with 4 mL of the appropriate solution, sealed with Parafilm, and incubated at 25 °C in the dark.

Germination measurement began on day 3 and continued to day 7 of incubation. Seeds with a radicle length of 2 mm or longer were considered germinated. Germination percentage (GermPer) was calculated as the percentage of seeds germinated within seven days relative to the total number of seeds per dish. On day 10, Petri dishes were removed from the incubator, and the seedling lengths (including the roots) were measured. Seedlings were then dried in a forced-air oven at 80 °C for 24 h before recording their dry weight. Mean seedling length (MLength) and mean dry weight (MWeight) were calculated for each replicate. For each replicate, seedling vigor index (Vigor3) was calculated as: Vigor3=GermPer×MLength×MWeight.

Overall, the seedling vigor test consisted of 774 accessions (including 16 accessions as control; Table S1) evaluated under three treatments with two replications each. Accessions were processed in sets of 24 and were run sequentially from July 2023 to November 2024.

### 2.3. Greenhouse Trial Under Salinity Stress

The salinity trial began with accession selection based on the PEG-based seedling vigor test, followed by greenhouse seed increase to generate sufficient seed for the trial. Although PEG-based seedling vigor index is not a precise measure of drought tolerance, it reflects an accession’s ability to maintain germination and seedling vigor under PEG-induced dehydration. Based on this reasoning, 95 accessions were selected from 774 assayed accessions: 89 accessions with the highest Vigor3 values under PEG20 and 6 accessions with exceptionally high germination rates under PEG10, despite no germination under PEG20. Four additional controls were employed, and they were selected from the 16-accession control set (Table S1): two Canadian cultivars (OAC Ayton and AAC Springfield) and two USA soybean accessions of Chinese origin (TMP29567 and TMP29568) previously reported to be drought tolerant [37]. The resulting 99 accessions were increased in the greenhouse at the Saskatoon Research and Development Centre, Agriculture and Agri-Food Canada, Saskatchewan, Canada. Seeds were planted in 6-inch pots containing Sunshine Mix #5 (Sun Gro Horticulture, Agawam, MA, USA) under conditions of 22 °C (day) and 18 °C (night), and a photoperiod of 16 h. The seed increase produced a total of 72 accessions (including four control accessions) with sufficient seed for salinity testing. These 72 accessions originated from 15 countries (Table S1).

The greenhouse salinity trial was carried out from September to December 2025 at the Salinity Tolerance Testing Facility (Salinity Lab; [38]), Swift Current Research and Development Centre, Agriculture and Agri-Food Canada, Saskatchewan, Canada. The greenhouse testing facility uses hydroponically nourished silica sand pots and has automatic control over irrigation, fertility, and root-zone salinity. The experimental design had 72 accessions, each with 18 plants, that were grown in 54 tanks under three levels of salinity stress (Table 1). Specifically, the salinity levels of the irrigation solution (EC_i_) were 1.2 dS m^−1^ (control nutrients only), 8.1 dS m^−1^ (moderate salinity), and 16.1 dS m^−1^ (severe salinity), along with a half-strength modified Hoagland solution [39] added to all tanks as baseline nutrition. These EC_i_ levels were chosen to represent the salinity variability present in the Canadian Prairies using the conventional relationship of soil EC_e_ and test solution EC_i_ where EC_e_ ≈ 0.5 EC_i_ [40], as they were comparable with those in soil conditions (EC_e_) as 0.6 dS m^−1^, 4 dS m^−1^, and 8 dS m^−1^ [41]. The 54 tanks were arranged as 6 replications of 9 tanks each. Within each replication, there were 3 tanks for each salinity level. The tanks were split into 24 sub-units, and each sub-unit was randomly assigned a plant accession number (Figure S1).

Each growth tank had a height of 1.0 m, diameter of 0.95 m, a growing surface area of 0.57 m^2^, and contained approximately 725 L of washed 99.8% pure silica sand with a bulk density of 1500 kg m^−3^. Each tank was irrigated from a separate solution tank in the basement of the Salinity Lab, allowing hydroponic solutions to be individually mixed and controlled for each growth tank throughout the test. At saturation, the sand retained a mean volumetric water content of 31.3%. The salt solutions were prepared according to the values in Table 1. Irrigation was automatically controlled to impose root-zone salinity at field capacity. At the time of seeding, the seed was dipped in a mixture of 95% reverse osmosis (RO) water and 5% sodium hypochlorite, and then rinsed with RO water to reduce seed-borne pathogens. Using a 48-plant spacing template, planting holes were created with a sand-seeding tool. Two seeds of each accession were placed in adjacent holes, the holes were covered with sand, and irrigation cycles were initiated. The tanks were flooded with the selected solutions four times a day and each irrigation continued for 5 min until the sand was completely saturated, and then the solutions were permitted to drain into the supply tanks for the next irrigation. Water lost by evapotranspiration was replenished with RO water weekly to maintain the concentrations of salts in solution. Hydroponic solutions were drained approximately six weeks after seeding and then replenished using the same procedure as the initial setup to maintain sufficient plant nutrition and water quality throughout the experiment. The greenhouse conditions were set to 24 °C (day) and 18 °C (night) temperatures and a relative humidity of 35–40%. A 16 h day/8 h night photoperiod was applied for the first 8 weeks, after which a 12 h photoperiod was used until the end of the test. Along with changes to the photoperiod, the daytime temperature was increased by 1 °C per week until reaching a temperature of 30 °C.

Data collection began with the daily recording of first seedling emergence, starting on day 4 after seeding and continuing to day 45. If two seedlings for an accession emerged in a tank, one was removed to obtain 24 plants per tank. Continuous monitoring of plant growth, along with recording various morphological traits, allowed for a comparative assessment of growth patterns under different salinity levels. For each plant, the number of days from seeding to the first flowering at stage R1 [42] was recorded. The number of days from seeding to maturity were also recorded, as well as plant survival at stage R8. At maturity, pods were collected and counted for each plant, and the number of seeds per pod and the total seed weight per plant were recorded. Following seed harvest, plant roots were dug out and photographed. The tap root was located and pulled straight to measure its length for each plant. These efforts generated a data set of seven traits per plant for a comparative analysis of soybean salinity tolerance: the number of days for seedling emergence (DaysE), the number of days to flowering (DaysF), the number of days to maturity (DaysM), the length of the tap root (RootL) in millimeters, the total number of pods (PodsN), the total number of seeds (SeedsT), and the total seed weight (or yield; SeedY) in grams.

### 2.4. Statistical Analysis

The collected data from the PEG-based seedling vigor test and the salinity test were first subjected to trait distribution analysis and summary statistic generation. It was found that the collected data for many traits was not normally distributed. To minimize variance heterogeneity for ANOVA, data transformations were performed on some trait data. PEG-based data for MWeight, MLength and Vigor3 were log-transformed using the log1p() function in R [43] to reduce right skewness and accommodate zero values. Similarly, the salinity-based data for PodsN, SeedsT, and RootL were transformed using log1p(). However, the salinity-based data for DaysE, DaysF, and SeedY were transformed using the Yeo–Johnson power transformation with R package bestNormalize. The transformation parameter (λ) was estimated separately for each trait, and the transformation was applied using the predict() function. All the original and/or transformed trait data from PEG-based and salinity tests were further subjected to three-factor ANOVA analysis using a custom R script with the dplyr package to determine the statistical significance of Accession, Treatment, and Replication, along with their two-way interactions. The proportion of total variance explained by each factor and each interaction was also calculated. These analyses allowed for a comparative assessment on the impacts of data transformation. Similarly, the pairwise trait correlations were also analyzed for all the original and/or transformed trait data for PEG-based and salinity tests and plotted using custom R scripts for ease of interpretation.

To make stress tolerance measurements more informative, we applied a new measure of relative tolerance (RT), calculated for each trait as the response under a stress level relative to the control, using data from both the PEG-based and salinity tests. Specifically, RTx is defined as *X*_2_/*X*_1_, where *X*_2_ and *X*_1_ are two random variables representing the same trait *X* being measured for a plant under a stress level and under a control level, respectively. The approximate mean and variance of RTx for the trait *X* for all plants of an accession can be obtained using Taylor series expansions [44] as below:

$$E[X_{2}/X_{1}]\approx \frac{{\bar{X}}_{2}}{{\bar{X}}_{1}}\;\mathrm{and}$$

$$Var(X_{2}/X_{1})\approx \frac{{\bar{X}}_{2}^{2}}{{\bar{X}}_{1}^{2}}(\frac{Var(X_{2})}{{\bar{X}}_{2}^{2}}+\frac{Var(X_{1})}{{\bar{X}}_{1}^{2}}-\frac{2Cov(X_{2},X_{1})}{{\bar{X}}_{2}{\bar{X}}_{1}}).$$

With the estimated variance, the mean estimate of RTx can be tested for significance from zero using a *t*-test. A custom shell script was specifically written to generate RT data for different traits from the original and/or transformed PEG-based and salinity data sets, to calculate their means and variances, and to test for significance from zero for an accession. The generated RT data sets were also subjected to trait distribution and three-factor ANOVA analyses.

### 2.5. Identifying Germplasm with Tolerance to Dehydration and Salinity Stresses

Based on RT values for Vigor3 under PEG20 relative to PEG0 in the PEG-based seedling vigor test, a set of soybean accessions with the highest tolerance to PEG-induced dehydration was identified. Similarly, accessions with the highest salinity tolerance were identified using the highest RT values for SeedY under severe salinity relative to the control salinity level. The selection considered only the RT values for Vigor3 and SeedY, as these two traits best represent seedling growth and yield performance in the two tests.

## 3. Results

### 3.1. PEG-Based Seedling Vigor Test

**Figure 1:**
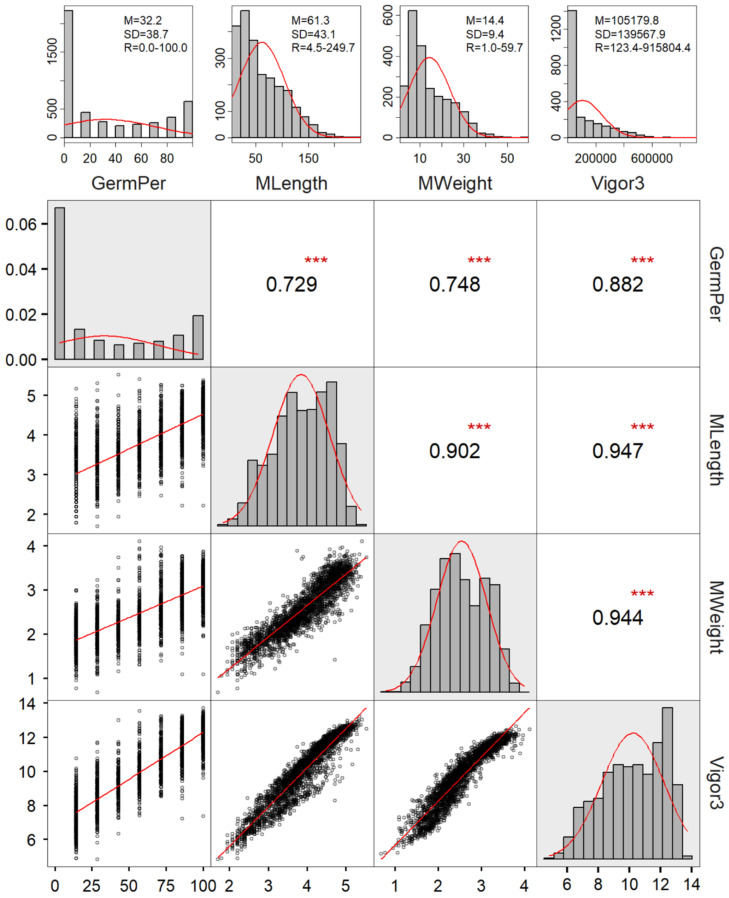
The frequency distributions and correlations of four traits (GermPer, MLength, MWeight and Vigor3) measured from the PEG-induced dehydration testing on 739 soybean accessions. The upper panel shows the frequency distributions of four traits without data transformation, along with the summary statistics (M = mean, SD = standard deviation, R = range). The lower panel displays the density distributions (in diagonal) and correlations of four traits with the data transformation. The upper diagonal shows the estimated correlation coefficients with the test of significance (*** for *p* < 0.001), and the lower diagonal displays the correlation patterns.

Out of 774 accessions tested, 32 had no germination in two replications, and 40 had germination in only one replication under the control level PEG0. Some germinations did not produce surviving seedlings for measurement. Effectively, there were only 739, 492, and 96 accessions with seedling measurements in one or two replications for PEG0, PEG10, and PEG20, respectively. Analyzing the collected data for four traits (GermPer, MLength, MWeight, Vigor3) revealed that the data for these traits did not follow normal distributions (Figure 1). Effort was made to transform these trait data for trait correlation analysis and ANOVA, except for GermPer data, as its arcsine square root transformation yielded little improvement in normality. The correlation analysis revealed high positive correlations among these four traits, with the estimates of correlation coefficient ranging from 0.729 to 0.947 (Figure 1). Three-factor ANOVAs of transformed and non-transformed trait data revealed highly significant effects for Accession, Treatment, and Accession × Treatment interaction for these traits, as illustrated in Table 2 for Vigor3. For example, Accession accounted for a higher proportion of the total variation than Treatment: 45.7% vs. 34.9% in transformed Vigor3 data and 36.7% vs. 30.3% in non-transformed data (Table 2). Figure S2A clearly showed substantial impacts on Vigor3 by PEG20 relative to PEG0. Clearly, the data transformation revealed more variation among accessions than among PEG-based treatments, with smaller variation for their interaction when compared to those based on non-transformed data.

To quantify the tolerance to PEG-induced dehydration, we calculated relative tolerance (RT) for each accession based on Vigor3 under PEG10 and PEG20 treatments. The results are given in Table S1. There were 485 and 95 accessions having RT values for PEG10 and PEG20, respectively, and two of the 95 accessions for PEG20 had no RT values for PEG10. Note that some accessions had no standard deviations for RT values as trait data was available for only one replication. RT values for PEG10 ranged from 0.002 to 59.6, with a mean of 0.481. Values larger than one were observed in 28 out of the 485 accessions, probably reflecting the outliers due to experimental errors. In contrast, RT values for PEG20 ranged from 0.001 to 0.471, with a mean of 0.040, after excluding three accessions with RT values larger than 1.000 that were also considered outliers. Most of these 95 accessions with RT values for PEG20 were selected for greenhouse seed increase for the salinity test. Interestingly, there were no significant (*p* > 0.05) differences in RT values for either PEG10 or PEG20 between the accessions representing 758 PGRC accessions and the 16-accession control set, indicating that the control group did not outperform the test PGRC accessions in seedling vigor.

To enhance the utilization of conserved soybean germplasm, 32 accessions with the highest RT values based on Vigor3 under PEG20 are listed in Table 3. Their RT values ranged from 0.031 to 0.471, with a mean of 0.096, and were statistically significant (*p* < 0.05) from the overall mean of 0.040 and the mean (0.037) of 16 control accessions (Table S1). These accessions originated from nine countries and included five control accessions. Four accessions were early maturity groups, MG 00, and one accession was MG 0. Eight accessions originating from Canada and China had RT values larger than 0.100, and three accessions with the highest RT values were CN29798 (from China; RT = 0.471), CN107453 (from Canada; RT = 0.274), and CN29789 (from China; RT = 0.266).

### 3.2. Salinity Test

Evaluating the seed yield data from surviving plants showed that the severe salinity strongly affected plant survival (Table S2). Specifically, under control salinity, each accession is expected to have six surviving plants, but there were 71 accessions with 4 to 6 surviving plants and one accession with only one surviving plant. Under moderate salinity, 69 accessions had four or more surviving plants, two accessions had one or two surviving plants, and one accession had no surviving plants. In contrast, under severe salinity, 22 accessions had four or more surviving plants, 36 accessions had 1 to 3 surviving plants, and 14 accessions had no surviving plants.

**Figure 2:**
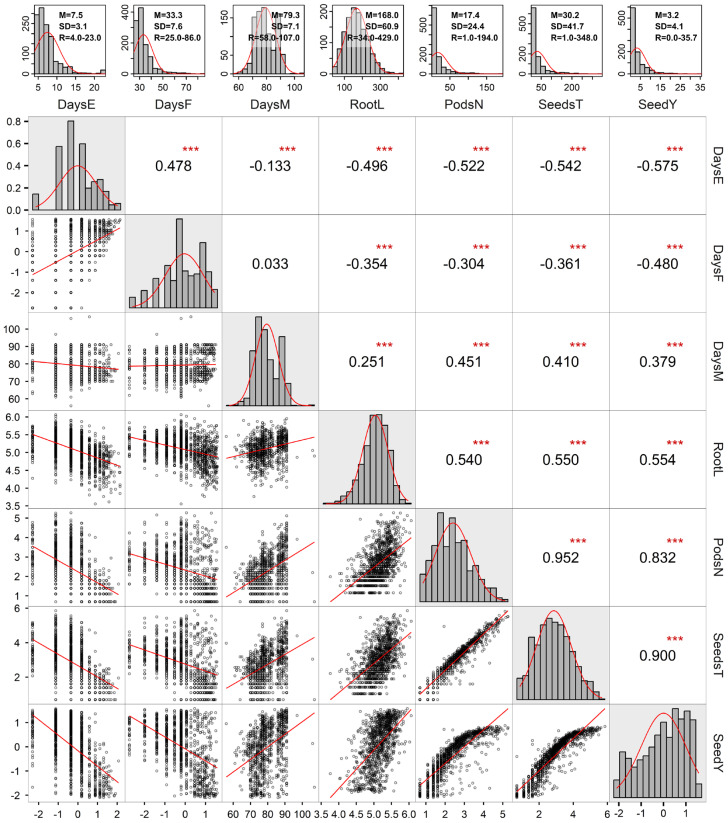
The frequency distributions and correlations of seven traits (DaysE, DaysF, DaysM, RootL, PodsN, SeedsT, and SeedY) measured from the salinity testing of 72 soybean accessions. The upper panel shows the frequency distributions of seven traits without data transformation, along with the summary statistics (M = mean, SD = standard deviation, R = range). The lower panel displays the density distributions (in diagonal) and correlations of seven traits with the data transformation. The upper diagonal shows the estimated correlation coefficients with the test of significance (*** for *p* < 0.001), and the lower diagonal displays the correlation patterns.

**Figure 3:**
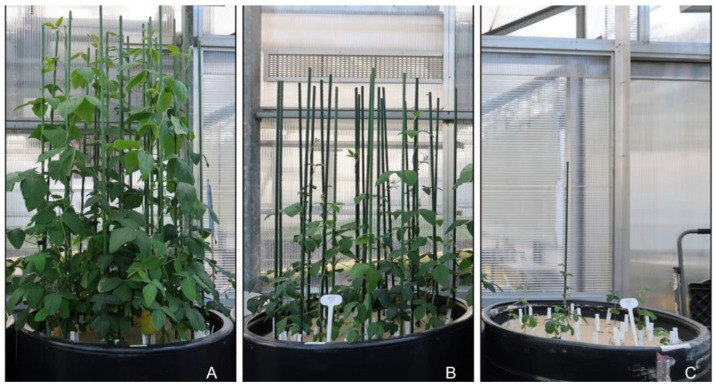
Illustration of comparative plant growth patterns of soybean germplasm under three salinity levels ((**A**) for EC_i_ 1.2 dS m^−1^; (**B**) for EC_i_ 8.1 dS m^−1^; (**C**) for EC_i_ 16.1 dS m^−1^) 44 days after seeding.

Analyzing the frequency distributions of seven traits measured during the salinity test revealed that only the data for DaysM showed an approximately normal distribution, while the other six traits displayed frequency distributions skewed to the right (Figure 2). Data transformations using two methods helped to generate approximately normal distributions for the six traits. Correlation analyses of both transformed and non-transformed data sets revealed significant and strong correlations among six of the seven traits, with the exception of the non-significant and weak correlation between DaysF and DaysM (Figure 2). Specifically, SeedY was strongly correlated positively with SeedsT (*r* = 0.900), PodsN (*r* = 0.832), followed by RootL (*r* = 0.554) and DaysM (*r* = 0.379), but moderately correlated negatively with DaysF (*r* = −0.480) and DaysE (*r* = −0.575) (Figure 2). Three-factor ANOVAs of transformed and non-transformed trait data revealed highly significant effects for all three factors (Accession, Salt and Rep) and their two-way interactions, as illustrated in Table 2 for SeedY. For transformed data, however, only Salt and Accession explained large proportions of the variation (52.1% and 18.0%, respectively), while Rep and the three interactions each explained 8.4% or less. This was more evident for Salt, as illustrated in Figure S2B. Similar patterns of variation were also revealed from the ANOVA of non-transformed data, but lower effects by Salt and Accession were detected, while the interactions of Accession × Salt and Accession × Rep showed higher effects (Table 2). These results indicate that the applied data transformations increased the proportion of total variation explained by Salt (52.1% vs. 20.6%) and decreased that explained by Accession (18.0% vs. 30.3%), compared to those based on non-transformed data. Higher effects by Salt than by Accession were consistent with the visual observations of plant growth during the salinity test (Figure S3 and Figure 3). The salinity impacts on plant growth were obvious at 30 days after seeding with more pronounced effects under severe salinity than moderate salinity (Figure S3). By 44 days after seeding, substantial impacts on soybean growth were observed. Clearly, soybean plants experienced substantial stress at both moderate and severe salinity levels (Figure 3B and Figure 3C, respectively) compared to the control treatment (Figure 3A).

Relative tolerance of each accession for these seven traits was calculated and presented in Table S3 for moderate salinity stress and in Table S4 for severe salinity stress. As shown in Table S3 for SeedY under moderate salinity, 71 accessions produced total seed yields per plant from 0.31 g to 8.64 g, with a mean of 2.11 g, and their RT values ranged from 0.05 to 0.89, with a mean of 0.39. Sixty-three (out of the 69 accessions with RT values) had RT values significantly (*p* < 0.05) greater than zero based on the *t*-tests. There were 16 accessions with RT values greater than 0.50 and three accessions with RT values larger than 0.84. On average, soybean plants had reduced total seed yield per plant from 5.41 g to 2.11 g under moderate salinity, corresponding to a mean RT value of 0.39. These RT values reflect the impact of moderate salinity on soybean growth and seed yield.

**Figure 4:**
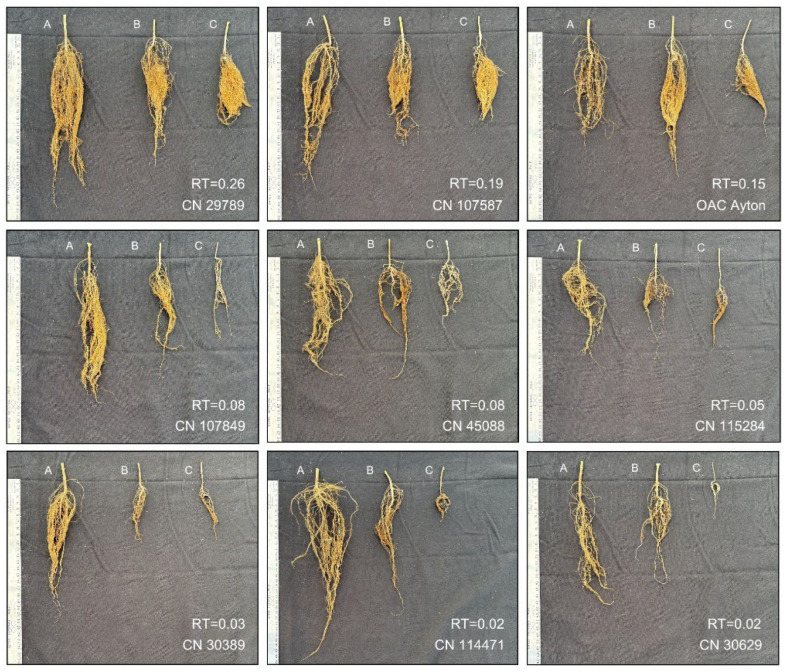
Illustration of comparative root patterns at harvest (in length and biomass) of nine soybean accessions under three salinity levels (A: for EC_i_ 1.2 dS m^−1^; B: for EC_i_ 8.1 dS m^−1^; C: for EC_i_ 16.1 dS m^−1^). Relative tolerance (RT) value for SeedY under severe salinity stress is also shown for each accession.

Under severe salinity stress (Table S4), 58 accessions produced total seed yields per plant from 0.03 g to 1.47 g, with a mean of 0.28 g. Analyzing the RT values for severe salinity revealed substantial impacts on soybean growth and seed yield. For example, soybean seedlings emerged by an average of 11 days after seeding, roughly 5 days later than plants under the control treatment (corresponding to a mean RT value of 2.00). Flowering started an average of 39 days after seeding, a delay of 9 days relative to the control treatment, with a mean RT value of 1.3. Consequently, the number of pods per plant decreased from 18.2 to 3.1 with a mean RT value of 0.17. The total seed yield per plant was reduced from 5.41 g to 0.28 g with a mean RT value of 0.13. The accession CN29789, with the highest RT value of 0.26, had a seed yield of 0.87 g (Table S4). To illustrate the high RT values obtained, nine accessions spanning low-to-high RT values were selected, and their root lengths and biomass under the three salinity levels are displayed in Figure 4. Clearly, the accessions with higher RT values had better root growth, with more roots and longer roots than those accessions with lower RT values. An extra correlation analysis revealed significantly positive correlations between RootL and SeedY RT values under moderate and severe salinity (Figure S4A,B). Interestingly, the two accessions of Chinese origin that were acquired from USDA as part of the control set produced no seeds, and the two other control accessions (OAC Ayton, AAC Springfield) had RT values of 0.15 and 0.04 (respectively) with a mean of 0.094, which was not statistically significant (*p* = 0.617) from the mean RT value of 0.068 for the other 56 accessions. However, OAC Ayton still provided a valuable control for comparison.

Further correlation analyses revealed two interesting findings. First, there was non-significant (*p* = 0.079) correlation between SeedY RT values under moderate and severe salinity (Figure S4C). Second, there were no correlations among Vigor3 RT values under two PEG-induced dehydration treatments (PEG10 and PEG20) and SeedY RT values under moderate and severe salinity (Figure S5). These findings implied that the assayed soybean plants had responses varying with the type and extent of abiotic stress.

To identify the most salt-tolerant accessions, two sets of 20 soybean accessions with the highest RT values under moderate and severe salinity stress are presented in Table 4. Under moderate salinity, the top 20 accessions had RT values ranging from 0.462 to 0.886, with an average of 0.605, and these RT values were statistically significant (*p* < 0.0001) from the overall mean of 0.390 and the mean (0.343) of four control accessions (Table S3). Nineteen accessions had RT values significantly greater than zero. There were two accessions with MG 0, six accessions with MG 00, and one accession with MG 000. These 20 accessions originated from 10 countries. Under severe salinity, the top 20 accessions had RT values ranging from 0.079 to 0.262 and averaging 0.130, and these RT values were statistically significant (*p* < 0.0001) from the overall mean of 0.069 but not significant (*p* = 0.310) from the mean (0.094) of four control accessions (Table S4). Nine accessions had RT values significantly greater than zero. There were two accessions with MG 0, eight accessions with MG 00, and one accession with MG 000. These 20 accessions originated from 11 countries. Notably, six accessions from five countries displayed higher RT values than OAC Ayton, the Canadian salt-tolerant cultivar, but OAC Ayton still had the highest seed yield (Table 4). Furthermore, only seven accessions were consistently identified in the top 20 accessions under both moderate and severe salinity tests (Table 4). Accession CN29789 originating from China held the highest RT values of 0.89 and 0.26 in both sets of salt-tolerant germplasm.

## 4. Discussion

Characterizing 774 soybean accessions through PEG-based seedling vigor and salinity tests identified two sets of soybean germplasm with tolerance to PEG-induced dehydration (Table S1 and Table 3) and salinity stress (Tables S3 and S4 and Table 4). The PEG-based seedling vigor test identified 95 accessions with vigorous seedling growth in Petri dishes containing 20% (*w*/*v*) PEG 6000 solution (Table S1). The salinity test revealed 58 accessions that produced total seed yields per plant from 0.03 g to 1.47 g under severe salinity (EC_i_ 16.1 dS m^−1^) (Table S4). Six accessions were found to display higher salt tolerance than the Canadian salt-tolerant cultivar OAC Ayton. Accession CN29789 was unique, as it consistently showed high tolerance to both dehydration and salinity stresses and had healthy root growth under severe salinity (Figure 4). Note that CN29789 was recorded as a soybean cultivar ‘Hei Nung No. 18’ (or ‘黑农18’ in Chinese) that was developed by the Heilongjiang Academy of Agricultural Sciences and presented to the visiting Canadian delegation of agricultural scientists in June 1974. These findings are significant, as they not only provide valuable germplasm for effective soybean breeding for abiotic stress tolerance but also demonstrate the value of characterizing plant germplasm conserved in a genebank for better utilization.

Earlier studies have demonstrated that PEG 6000 can be used as an osmotic agent to simulate water deficit without causing toxic effects on plant growth [45,46]. A 20% PEG 6000 solution (PEG20) should impose significant osmotic stress on plant growth (e.g., see [47]). Our PEG-based test revealed only 95 (out of 774) accessions with RT values for Vigor3 under PEG20 relative to PEG0. The RT values of these 95 accessions ranged from 0.001 to 0.471, with a mean of 0.040. These results clearly demonstrated the impact of PEG-induced dehydration on seed germination and seedling growth. Encouragingly, there were 32 accessions displaying considerable tolerance to PEG20 with RT values greater than 0.03, and three accessions (CN29798, CN107453, CN29789) with the highest RT values (Table 2). However, our PEG-based test also carried some weakness. With limited seed availability from the PGRC soybean collection, only seven seeds per dish were assayed per replication and only two replications were conducted. This limitation generated larger variability in germination percentage (GermPer) and consequently in Vigor3. It also resulted in many accessions with RT values based on only one replication, which limited our ability to test the significance of their RT values. Future PEG-based tests could proceed with only two PEG treatments, such as PEG0 vs. PEG20, so that more seeds per dish would be possible and more replications could be applied. Alternatively, more efficient laboratory-based tests need to be explored for characterizing a large number of plant accessions conserved in genebanks.

Our greenhouse salinity test had six replications and a well-balanced growth tank layout for 72 accessions under three salt treatments and was successful in the assessment of salt tolerance for these soybean accessions (Tables S3 and S4). In this test, we focused on the salinity impact on soybean growth and yield and did not measure relevant physiological traits such as stomatal conductance, chlorophyll fluorescence, and proline content, thus limiting the inference of the mechanisms underlying soybean salinity tolerance. Although all three factors and three two-way interactions were statistically significant, the significant proportions of the total variation explained by Rep and the three two-way interactions were much smaller than those by Salt and Accession (Table 2), and consequently, should not affect our interpretation of the effects by Salt and Accession, as illustrated with the ANOVA results from transformed data (Table 2). However, as our selected salinity levels may not fully reflect the salt variability in the Canadian Prairies [41], a field test across the Canadian Prairies of the elite soybean accessions identified from this salinity test [48] would still be desirable to validate the reported salt tolerance. The field test could proceed under either natural or imposed salinity, with only a few accessions having the highest RT values for SeedY under severe salinity (Table 4).

Our characterization focused on soybean tolerance to dehydration and salinity stresses, two different aspects of soybean physiological drought [49,50]. Some accessions such as CN29789 consistently showed high tolerance to both dehydration and salinity stresses. However, some accessions showed good tolerance only to one stress, such as CN29798 for PEG-induced dehydration and CN107490 for salinity stress. Such differences may have reflected the true differences in physiological drought or some experimental errors during the PEG-based and/or salinity tests, as suggested in Figure S5, which shows no significant correlations with respect to stress and trait. Our tests were not designed to explore and understand the physiological mechanisms of tolerance to dehydration and salinity stresses [22,51], but our findings support research in this area by providing preliminary evaluations of these soybean accessions [52]. The accession CN29789 would be the study material of choice to investigate the molecular mechanisms responsible for high tolerance to both dehydration and salinity stresses.

The soybean accessions identified with high tolerance to dehydration and salinity stresses provide a new set of valuable soybean germplasm for effective soybean breeding. Three accessions (CN29798, CN107453, CN29789) exhibited strong tolerance to 20% PEG 6000 solution (Table 3). Six accessions (CN29789, CN107808, CN107490, CN107587, CN107873, CN35757) showed stronger tolerance to severe salinity than OAC Ayton, although OAC Ayton still had the highest seed yield (Table 4). These six accessions displayed vigorous and healthy root growth under severe salinity (Figure 4), a characteristic commonly associated with enhanced drought tolerance. Some studies have demonstrated that a deep, vigorous root system with extensive lateral branching can enhance plants to extract water from larger soil volumes (e.g., see [26,53,54]). Also, some highly stress-tolerant accessions were early-maturing (MG 000 to MG 0; Table 3 and Table 4), showing an added adaptation to the Canadian Prairies. Thus, these stress-tolerant accessions, along with those core subsets identified from previous germplasm characterizations on early maturity, high oil, and protein concentration [36], will form a package of valuable soybean germplasm for smart soybean breeding [55] to develop new productive cultivars that are resilient to the soil salinity and changing climate in the Canadian Prairies [18,19]. The need for cultivars with abiotic stress tolerance is increasingly urgent as higher temperatures and extreme weather events have become more frequent [56], and soil salinization is a significant threat to agricultural soils and water resources in the Prairie provinces [20,21].

The reported findings also have implications for the management of soybean germplasm conserved in a genebank. First, this characterization effort has generated a new set of characterization data on tolerance to dehydration and salinity stresses for the conserved germplasm, and identified 32 soybean accessions with no germination. This data set can be integrated into a genebank database such as GRIN-CA for public access. For example, the reported soybean accessions with tolerance to dehydration and salinity stresses can be acquired for research and breeding via PGRC germplasm requests (https://pgrc-rpc.agr.gc.ca/gringlobal/landing; accessed 6 March 2026). Also, the data could be useful for setting up management priorities to conserve the germplasm, such as timely viability testing and effective regeneration. Second, the successful identification of soybean germplasm with tolerance to dehydration and salinity stresses not only confirmed the value of conserving soybean genetic diversity in a genebank, but also demonstrated the need to characterize conserved germplasm for better utilization [8]. As discussed above, this characterization effort, along with several others [36], have yielded multiple sets of elite soybean germplasm that can enhance soybean breeding [2]. Thus, greater efforts are needed to identify adapted and valuable germplasm conserved in genebanks to accelerate breeding for crops with better resilience to environmental and climate risks [10,57]. This search could be carried out more effectively through funded collaborative research involving genebank stakeholders such as plant researchers, breeders, and industries [7,8].

## 5. Conclusions

This germplasm characterization has generated two sets of soybean accessions with tolerance to PEG-induced dehydration and salinity stresses. The PEG-based seedling vigor test identified 95 accessions with seedling growth in Petri dishes containing 20% (*w*/*v*) PEG 6000 solution. The salinity test revealed 58 accessions that produced total seed weight per plant from 0.03 g to 1.47 g under severe salinity. Six accessions originating from five countries had higher salt tolerance than the Canadian salt-tolerant cultivar OAC Ayton, but the latter still had the highest seed yield. One unique accession, CN29789, originating from China with the name of ‘Hei Nung No.18’, consistently showed high tolerance to both water and salinity stresses and had healthy root growth under severe salinity stress. These findings are significant, as they not only provide useful germplasm for soybean breeding on abiotic stress tolerance but also demonstrate the value of characterizing plant germplasm conserved in a genebank for better utilization.

## Figures and Tables

**Table 1:** Properties of the three salt treatments employed for salt tolerance in a tank of 72 soybean accessions. Baseline nutrition is provided as a modified Hoagland solution. Salt amounts shown in the table are supplemental to the modified Hoagland solution. The NaCl concentration is given as an average across the growth tanks. EC_i_ is the electrical conductivity of the complete solutions.

Treatment	Baseline Nutrition	CaCl_2_(g L^−1^)	MgSO_4_(g L^−1^)	Na_2_SO_4_ (g L^−1^)	NaCl (g L^−1^)	EC_i_ (dS m^−1^)
Control	Included	0	0	0	0	1.2
Moderate	Included	1.019	0.503	2.639	1.192	8.1
Severe	Included	1.869	0.988	8.364	1.910	16.1

**Table 2:** ANOVA results on transformed data of Vigor3 acquired by the PEG-based testing of 739 accessions with two replications under three levels of dehydration and on transformed data of SeedY acquired by the salinity testing of 72 accessions with six replications under three salinity levels. The proportions of the total variance explained by three factors and their two-way interactions are also given for ANOVAs performed on transformed and original collected data sets to illustrate their comparative effects.

Sources of Variation	Degree of Freedom	Sum of Squares	Mean Square	F Value	Pr (>F)	Variance (%) for Transformed Data	Variance (%) for Original Data
**PEG-based testing**							
Accession	738	4377	5.90	9.56	<2 × 10^−16^	0.457	0.367
Treatment	2	3341	1670.40	2691.56	<2 × 10^−16^	0.349	0.303
Rep	1	0	0.50	0.80	0.3712	0.000	0.000
Accession:Treatment	579	1147	2.00	3.19	<2 × 10^−16^	0.120	0.190
Accession:Rep	696	499	0.70	1.16	0.0639	0.052	0.092
Treatment:Rep	2	1	0.70	1.19	0.3047	0.000	0.000
Residuals	347	215	0.60			0.022	0.048
**Salinity testing**							
Accession	71	180.6	2.54	13.41	<2 × 10^−16^	0.180	0.303
Salt	2	524	262.01	1381.12	<2 × 10^−16^	0.521	0.206
Rep	5	23.4	4.67	24.64	<2 × 10^−16^	0.023	0.027
Accession:Salt	127	64.3	0.51	2.67	4 × 10^−14^	0.064	0.098
Accession:Rep	347	83.5	0.24	1.27	0.0092	0.083	0.174
Salt:Rep	10	45.1	4.51	23.77	<2 × 10^−16^	0.045	0.022
Residuals	443	84	0.19			0.084	0.168

**Table 3:** The list of 32 soybean accessions with the highest values of relative tolerance (RT) for Vigor3 under PEG20 over the control PEG0, along with the standard deviations (RTsd) of their RT values and the summary statistics (SD = standard deviation, Ns = the number of samples) of non-transformed Vigor3 data for each accession under PEG20. MG is for early maturity group (2 for MG 00 and 3 for MG 0), NA is not available. Three RT values in bold indicate statistical significance larger than zero.

Accession	Origin	Description	MG	Mean	SD	Ns	RT	RTsd
*32 accessions*								
CN29798	CHN	Wu Ding Choo	NA	465.1	NA	1	0.471	NA
CN107453	CAN	Electron	NA	46,267.2	22,717.2	2	0.274	0.098
CN29789	CHN	Hei Nung No. 18	NA	482.3	NA	1	0.266	NA
TMP29567	CHN	PI 567690, Fu yang (7)	NA	51,566.6	42,386.4	2	0.164	0.126
CN107411	CAN	Micron	NA	295.7	NA	1	0.153	NA
CN107451	CAN	OT96-25	NA	4800.0	NA	1	0.151	NA
CN29792	CHN	Feng Shou No. 12	NA	477.7	NA	1	0.126	NA
CN114471	CAN	Kamichis	NA	6013.3	6576.7	2	**0.103**	0.016
CN35343	KOR	KAS362-5	NA	882.9	NA	1	0.098	NA
CN33266	CAN	Harcor	NA	1192.0	NA	1	0.091	NA
TMP29568	CHN	PI 567731, Fu yang (56)	NA	24,135.8	8615.6	2	0.091	0.027
CN52740	CAN	OT94-41	NA	726.1	NA	1	0.085	NA
CN35371	KOR	KAS643-1	NA	470.6	NA	1	0.073	NA
CN107412	CAN	AC Colibri	NA	220.6	NA	1	0.073	NA
CN107502	FRA	B 10	3	30,435.0	9251.0	2	**0.071**	0.011
CN115284	RUS	Saliut	NA	25,910.5	NA	1	0.070	NA
TMP29570	USA	PI 647960, R01-416F	NA	25,052.7	10,705.9	2	0.063	0.016
CN29793	CHN	Jao Teh Jar Ching	NA	795.1	NA	1	0.060	NA
CN30389	RUS	Amurskaja 042	NA	1575.0	NA	1	0.058	NA
CN107577	CHN	Shika No. 4	2	6811.9	NA	1	0.054	NA
CN107511	FRA	Jaune De Desme	2	15,494.1	NA	1	0.051	NA
TMP29565	USA	PI 471938, 197	NA	15,993.5	18,561.7	2	0.046	0.066
CN35757	UKR	Terezikskaja 24	NA	1702.9	NA	1	0.045	NA
CN44436	KOR	Kaeri-Gnt 641-3	NA	12,921.8	1141.9	2	0.040	0.037
CN107879	UNK	V-16	2	1085.1	NA	1	0.039	NA
CN45088	YUG	1015	NA	12,190.4	5892.6	2	0.038	0.041
CN35364	KOR	KAS629-1	NA	625.4	NA	1	0.037	NA
CN107580	HUN	Iregy soja	2	4811.4	NA	1	0.036	NA
CN107651	RUS	Salut 216 UDSSR	NA	9858.3	NA	1	0.035	NA
TMP29569	USA	PI 592756, Dillon	NA	14,828.7	3390.3	2	0.033	0.010
CN30633	HUN	Iregi Korona	NA	1319.6	837.0	2	**0.031**	0.006
CN39086	CAN	X702-3-2	NA	1382.6	702.6	2	0.031	0.016
*16 control accessions*								
Mean							0.037	0.043

**Table 4:** Two sets of 20 soybean accessions with the highest values of relative tolerance (RT) for SeedY under moderate (on the left) and severe (on the right) salinity over control salinity, along with the standard deviations (RTsd) of their RT values and the means of non-transformed SeedY data for each accession. MG is for early maturity group (1 for MG 000, 2 for MG 00, 3 for MG 0). NA is not available. RT values in bold indicate statistical significance larger than zero. Seven accessions in bold appeared in both sets.

Accession	Origin	Description	MG	Moderate Salinity			Accession	Origin	Description	MG	Severe Salinity		
				Mean	RT	RTsd					Mean	RT	RTsd
*20 accessions*													
**CN29789**	CHN	Hei Nung No. 18	NA	2.93	**0.886**	0.709	**CN29789**	CHN	Hei Nung No. 18	NA	0.87	**0.262**	0.136
CN39086	CAN	X702-3-2	NA	3.44	0.866	1.172	CN107808	FRA	Grignon 1	3	0.96	0.229	NA
CN107879	UNK	V-16	2	6.56	**0.854**	0.793	**CN107490**	DEU	Strain No. 14	2	0.47	**0.193**	0.053
CN32351	ROU	No. 850	NA	8.46	**0.762**	0.521	CN107587	CHN	PI 358321b	2	0.67	**0.188**	0.084
CN33247	CAN	Hardome	NA	3.18	**0.676**	0.340	**CN107873**	POL	Mlochowska	1	0.48	0.163	NA
**CN30318**	CHN	See Table S1	NA	3.07	**0.605**	0.239	CN35757	UKR	Terezikskaja 24	NA	0.57	**0.152**	0.064
**CN107474**	DEU	B-34	2	0.94	**0.589**	0.422	OACAyton	CAN	Cultivar	NA	1.47	0.146	0.270
CN30633	HUN	Iregi Korona	NA	5.57	**0.585**	0.464	**CN107474**	DEU	B-34	2	0.22	**0.137**	0.154
CN107590	RUS	Amurskaja 310	3	1.30	**0.576**	0.580	CN115283	DEU	See Table S1	NA	0.40	**0.126**	0.143
**CN39087**	CAN	X702-3-3	NA	1.21	**0.575**	0.413	CN107623	RUS	Staroukrainskaja	2	0.38	0.109	0.238
CN35344	KOR	KAS362-7	NA	2.92	**0.575**	0.529	**CN107582**	ROU	S-16	2	0.28	**0.106**	0.091
CN107375	CAN	Blackjack 21	NA	8.64	**0.548**	0.503	**CN30318**	CHN	See Table S1	NA	0.52	0.102	NA
**CN107582**	ROU	S-16	2	1.42	**0.539**	0.238	CN107862	DEU	No. 119-49	2	0.68	0.100	0.123
**CN107490**	DEU	Strain No. 14	2	1.27	**0.516**	0.262	CN107819	JPN	See Table S1	3	0.42	0.098	0.121
CN107511	FRA	Jaune De Desme	2	1.18	**0.515**	0.394	CN107451	CAN	OT96-25	NA	0.19	0.088	NA
CN35339	KOR	KAS351-4	NA	4.43	**0.511**	0.412	CN29795	CHN	See Table S1	NA	0.29	0.085	NA
**CN107873**	POL	Mlochowska	1	1.45	**0.494**	0.323	CN45088	YUG	1015	NA	0.47	**0.083**	0.070
CN107634	JPN	B44	2	2.38	**0.489**	0.469	CN107849	SWE	699-2-4	2	0.28	**0.082**	0.088
CN52736	CAN	OT94-49	NA	1.96	**0.480**	0.370	**CN39087**	CAN	X702-3-3	NA	0.17	0.081	0.104
CN107557	HUN	See Table S1	3	1.31	**0.462**	0.302	CN107469	CAN	Manitoba Brown	2	0.16	0.079	0.186
*4 control accessions*													
Mean					0.343	0.009						0.094	0.074

## Data Availability

Additional data presented in this study are available in Supplementary Materials.

## Supplementary Materials

The following supporting information can be downloaded at: https://doi.org/10.6084/m9.figshare.31852765. Table S1: Inventory data of 774 soybean accessions and values of relative tolerance (RT) to dehydration under PEG10 and PEG20 solutions, Table S2: Summary of 72 accessions with surviving, seed-yielding plants under three salinity levels, Table S3: The list of 72 soybean accessions with relative tolerance (RT) values for seven traits under moderate salinity over the control, along with the standard deviations of their RT values (RTsd) and the means of non-transformed data, Table S4: The list of 72 soybean accessions with relative tolerance (RT) values for seven traits under severe salinity over the control, along with the standard deviations of their RT values (RTsd) and the means of non-transformed data, Figure S1: Tank plan for the salinity testing of 72 soybean accessions with six replications under three salinity levels, Figure S2: Boxplots of transformed Vigor3 data for three PEG-induced dehydration treatments (A) and transformed SeedY data for three salinity levels (B) to illustrate the impacts of dehydration and salinity treatments, Figure S3: Illustration of comparative plant growth patterns of soybean germplasm under three salinity levels (A: for ECi 1.2 dS m^−1^; B for ECi 8.1 dS m^−1^; C for ECi 16.1 dS m^−1^) 30 days after seeding, Figure S4: Correlations of SeedY vs. RootL relative tolerance (RT) values under moderate (A) and severe (B) salinity, and of SeedY RT values between moderate and severe salinity (C), and Figure S5: Correlations of Vigor3 relative tolerance (RT) values under two PEG-induced dehydration treatments (PEG10 and PEG20) and SeedY RT values under moderate and severe salinity.
